# Supplementary material for: Multi-Strain and -Species Investigation of Volatile Metabolites Emitted from Planktonic and Biofilm Candida Cultures
Source: Metabolites. 2022 May 11;12(5):432. doi: 10.3390/metabo12050432 (PMC9146923; doi:10.3390/metabo12050432)
Supplement: Supplementary file 1 [file metabolites-12-00432-s001.zip › metabolites-1696776-supplementary.pdf]

Supplementary Information

Multi-Strain and -Species Investigation of Volatile Metabolites emitted from Planktonic and Biofilm *Candida* Cultures

Shane Fitzgerald [1], Ciara Furlong [2], Linda Holland [2], and Aoife Morrin [1*]

1 School of Chemical Sciences, National Centre for Sensor Research, Insight Science Foundation Ireland Research Centre for Data Analytics, Dublin City University, Dublin 9, Ireland
2 School of Biotechnology, Dublin City University, Dublin 9, Ireland

* Correspondence: aoife.morrin@dcu.ie

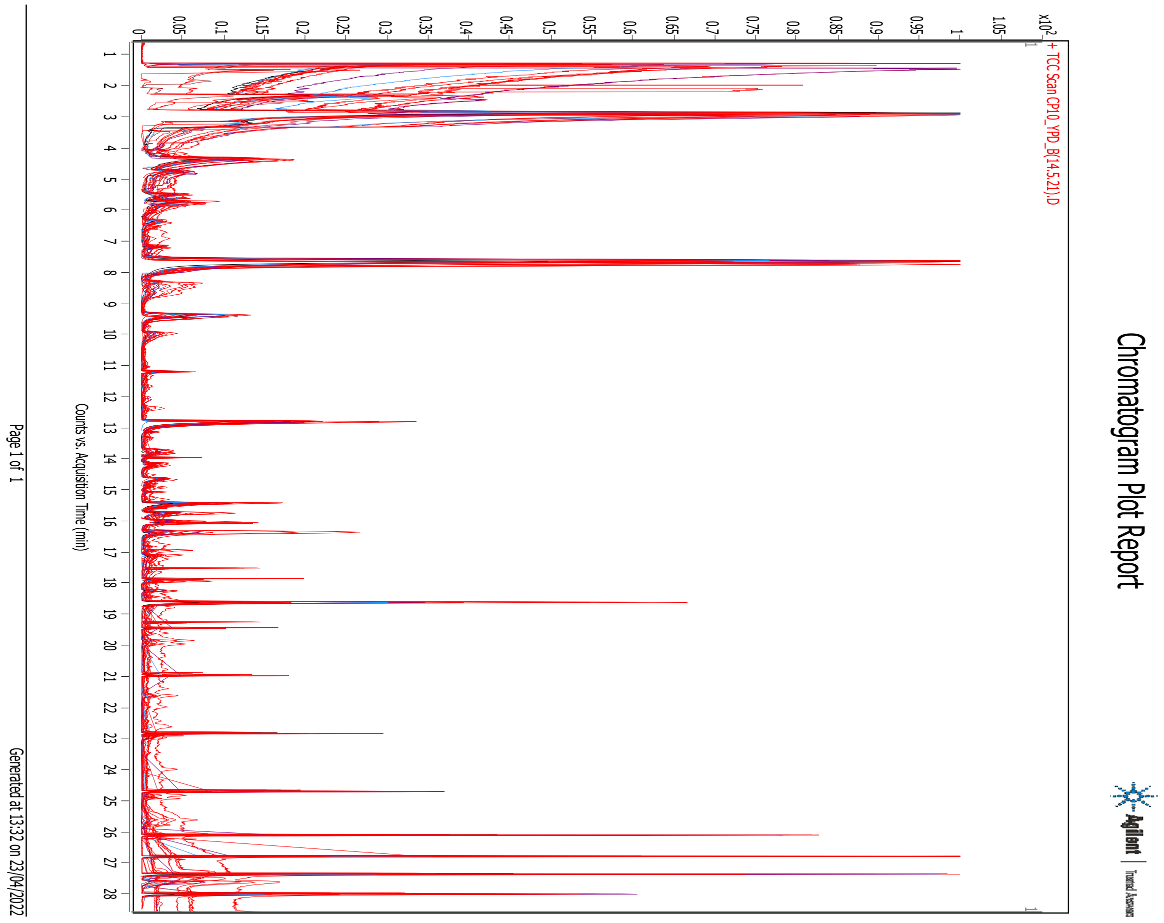

Figure S1. Overlayed chromatograms of planktonic *C. parapsilosis* strains 1-10 in YPD media.

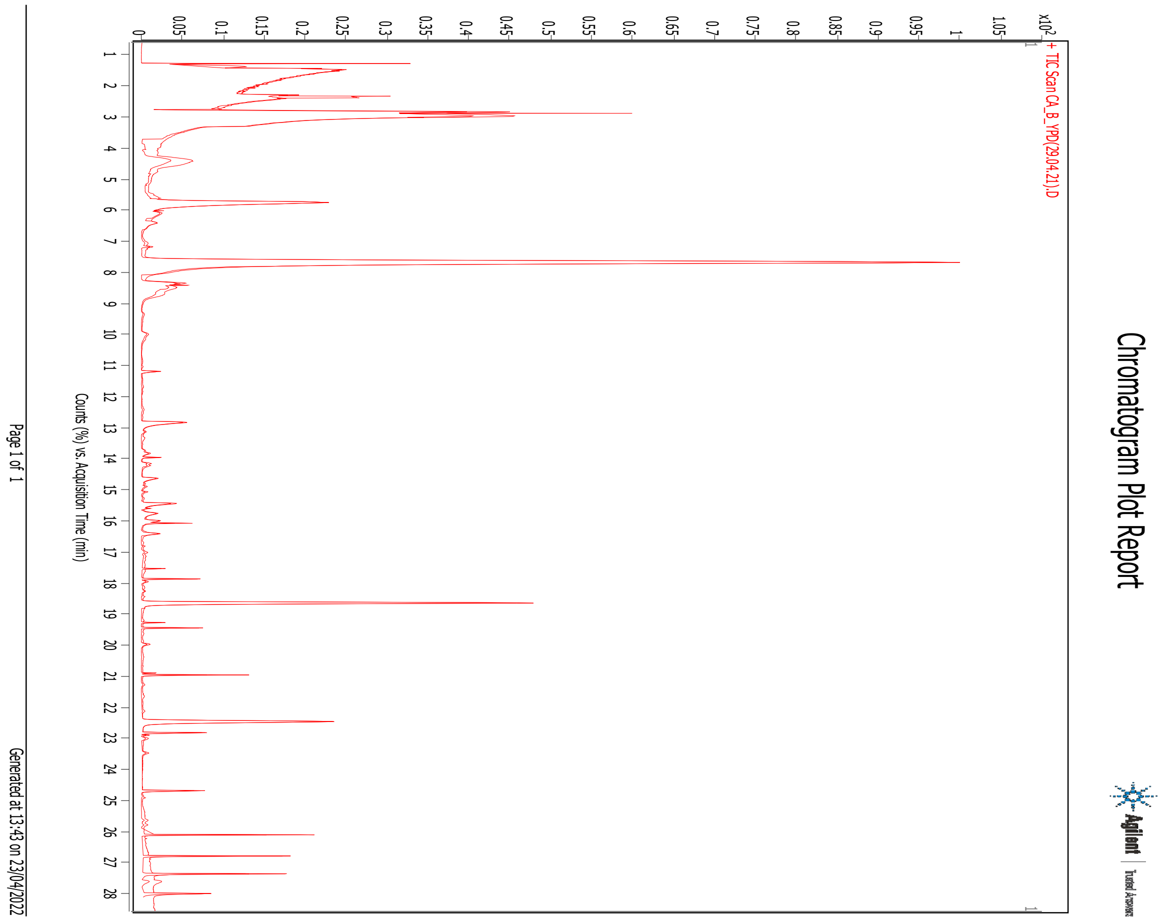

Figure S2. Chromatogram of planktonic *C. albicans* YPD culture.

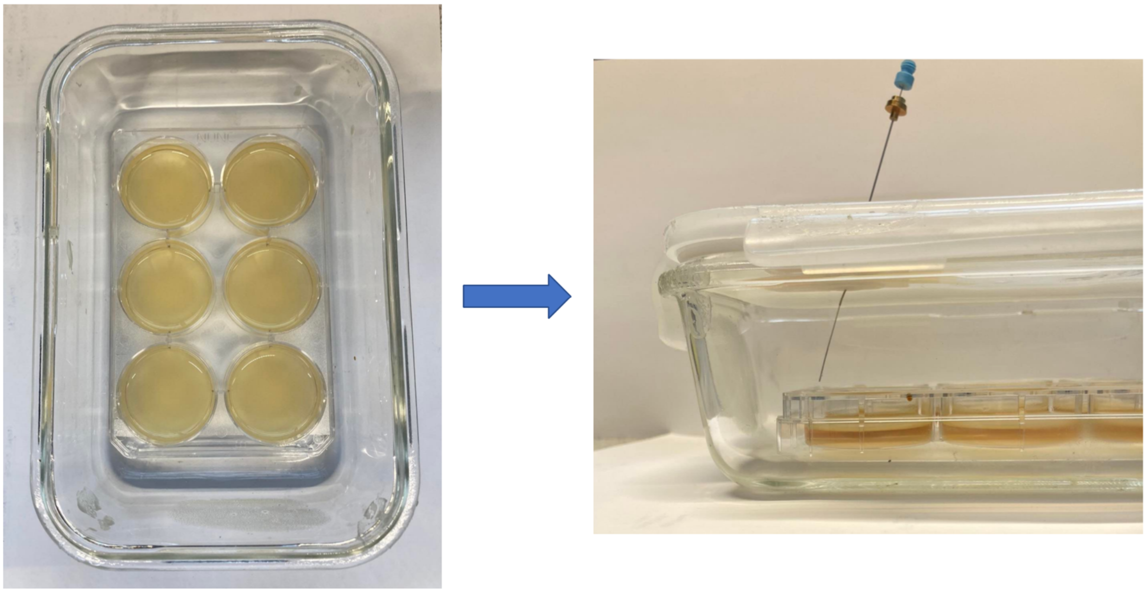

Figure S3. Experimental set-up for the SPME sampling of biofilm cultures. (Left) Top view of biofilm sample in container; (Right) Side view of SPME sampling of biofilm metabolites.

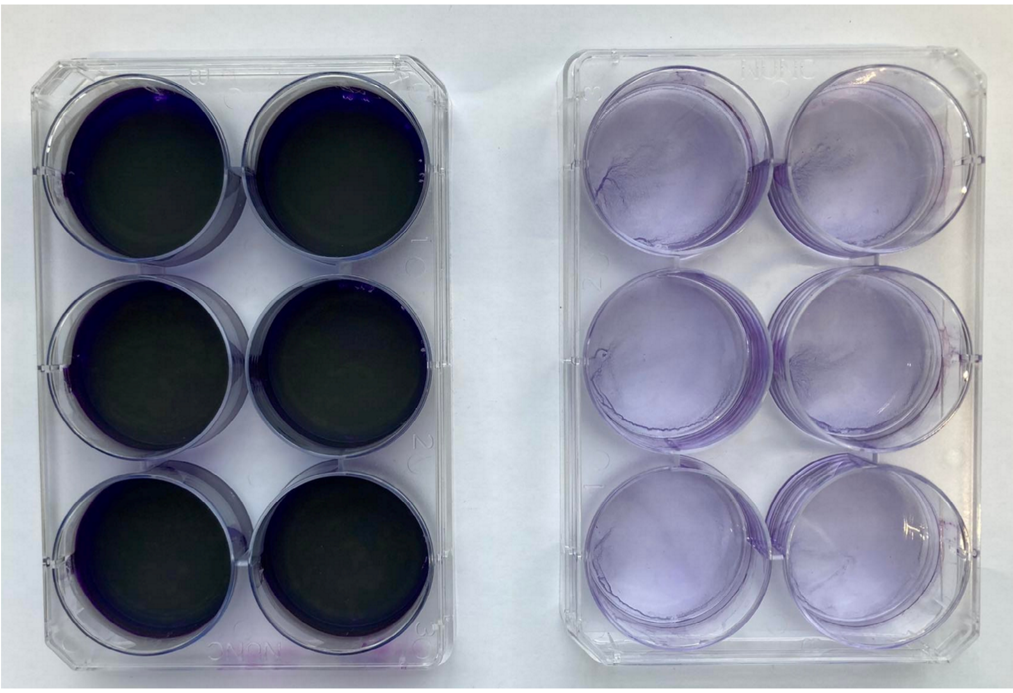

Figure S4. Crystal violet stain of biofilm-positive C. parapsilosis (CP6) samples (left) to verify biofilm formation; and biofilm-negative C. albicans samples (right).

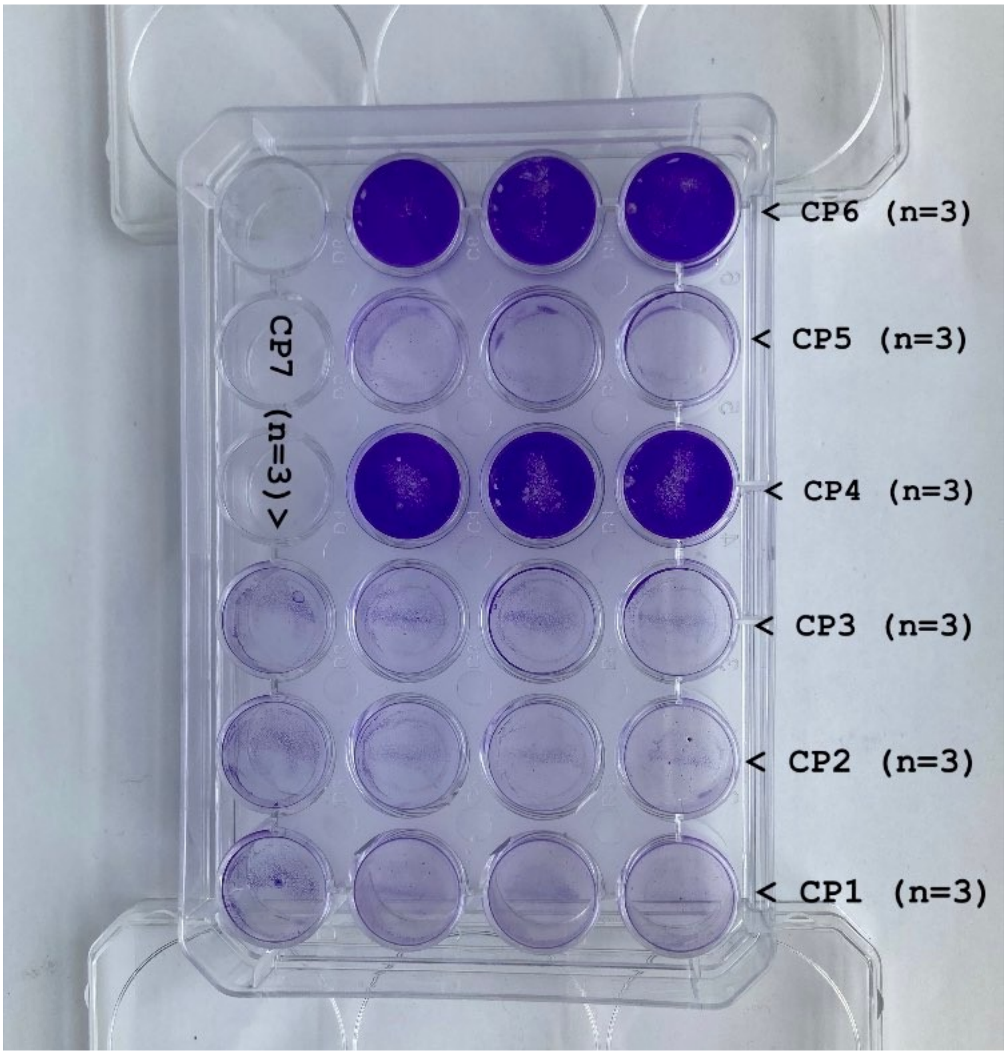

Figure S5. Crystal violet – based biofilm viability assay on CP1 – CP7. Out of the 7 CP strains tested, CP4 and CP6 were biofilm-positive.

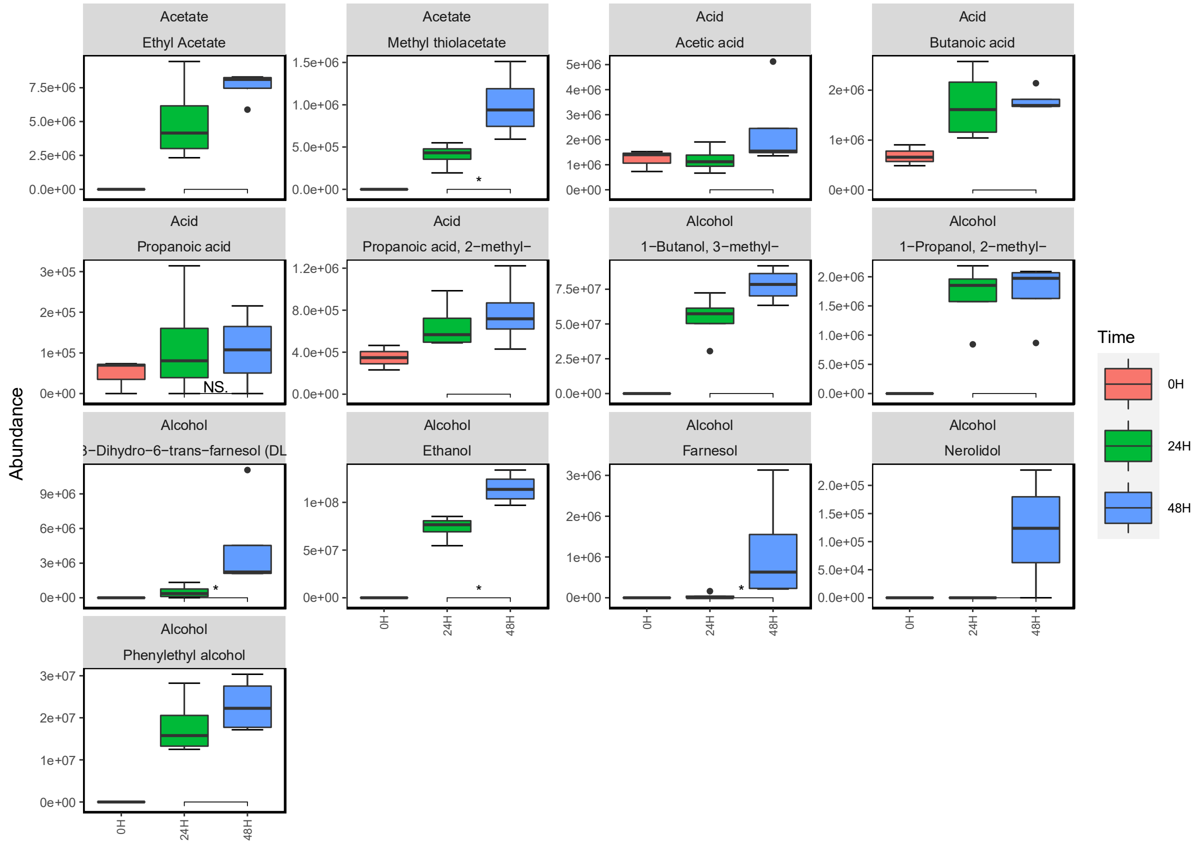

Figure S6. Compound boxplots illustrating the abundances of selected compounds detected in the headspace of biofilm-negative C. albicans cultures at 0, 24, and 48 h growth. Statistically significant differences in the abundance of compounds at each time point are illustrated through the star system where * = $p < 0.05$, ** = $p < 0.01$. Y-axis labels are displayed in scientific notation where ae+b = $a \times 10^b$.

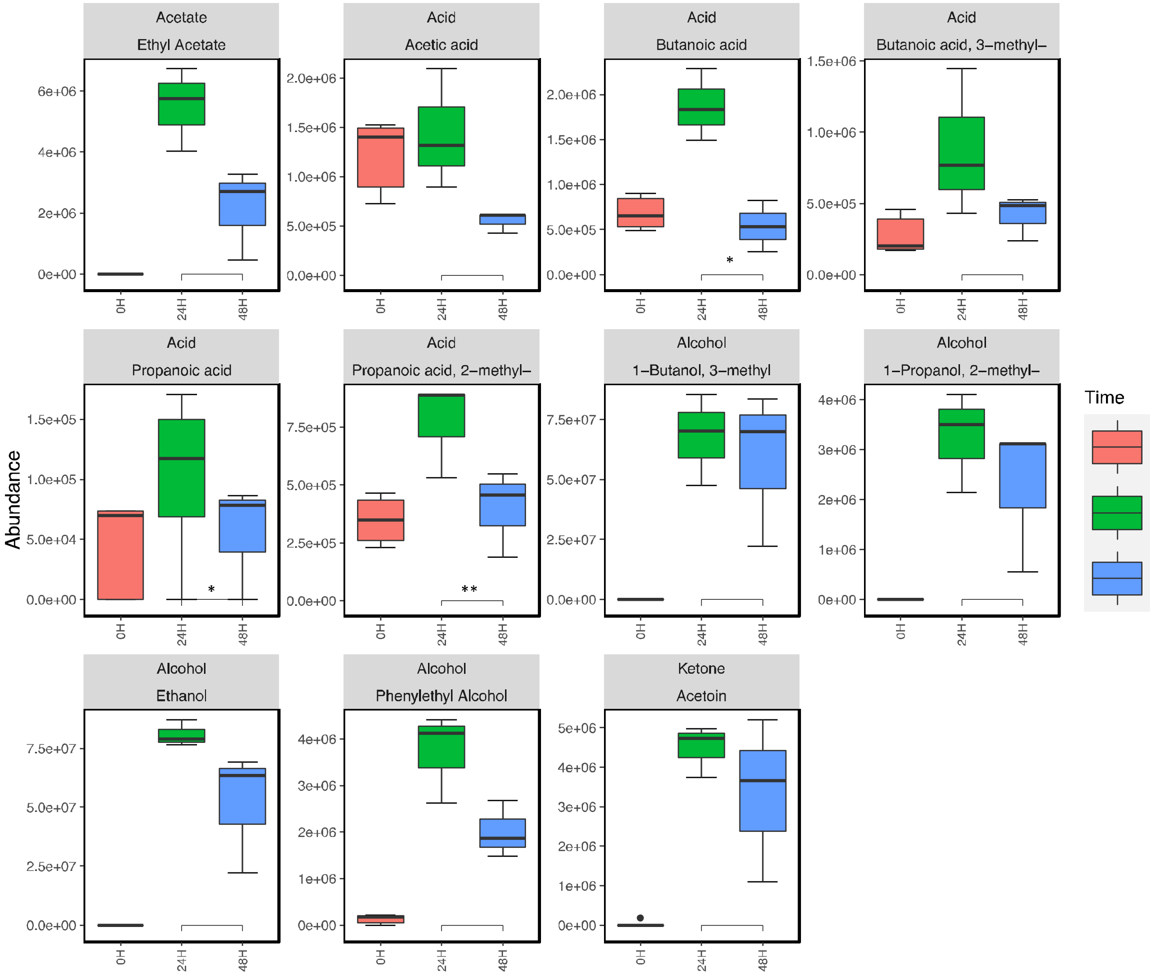

Figure S7. Compound boxplots illustrating the abundances of various compounds detected in the headspace of biofilm-negative CP1 cultures at 0, 24, and 48 h growth. Statistically significant differences in the abundance of compounds at each time point are illustrated through the star system where * = $p < 0.05$, ** = $p < 0.01$. Y-axis labels are displayed in scientific notation where ae+b = a × 10^b.

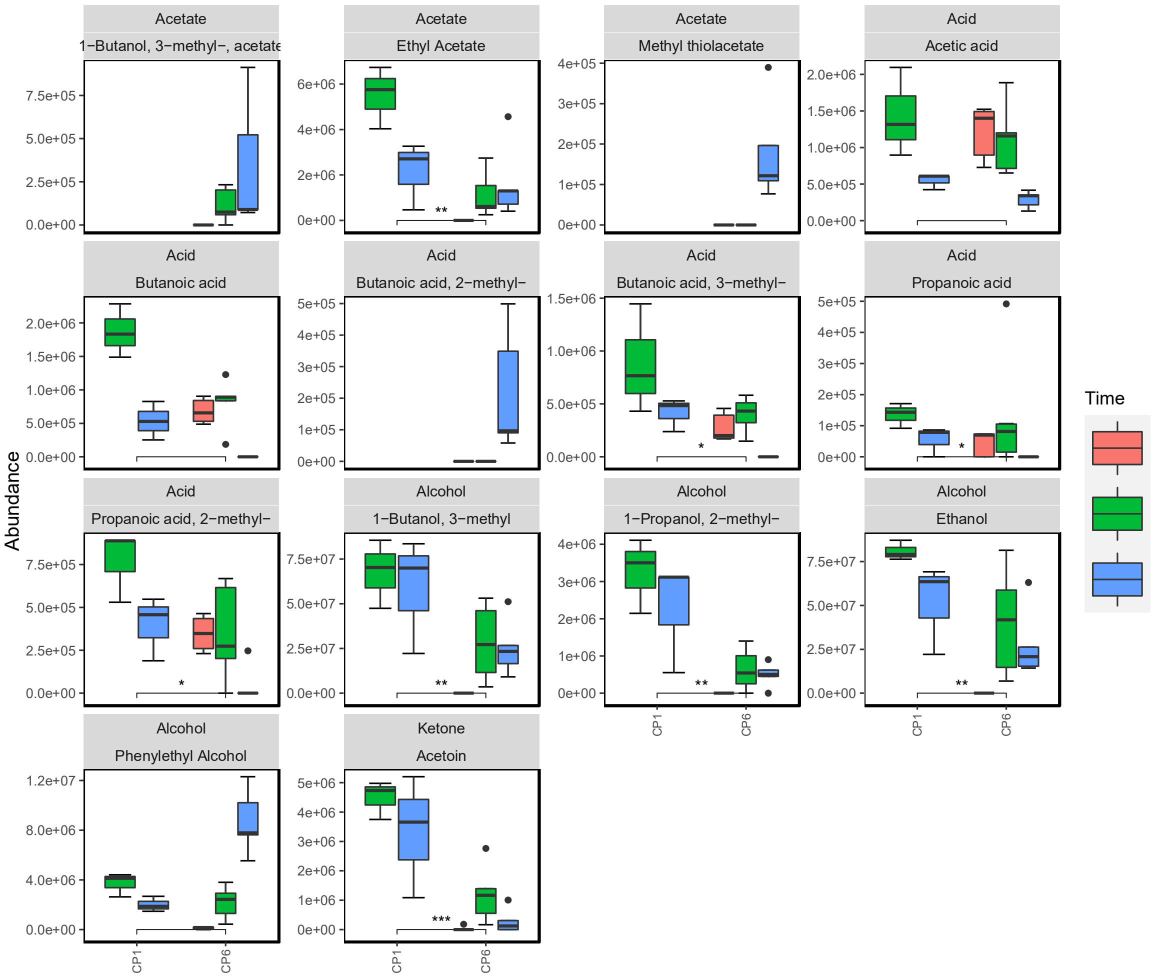

Figure S8. Compound boxplots illustrating the differences in abundances of various compounds detected in the headspace of non-biofilm forming CP1 and biofilm-forming CP6 cultures at 0, 24, and 48 h growth. Statistically significant differences in the abundances of each compound detected between the two strains are illustrated through the star system where * = $p < 0.05$, ** = $p < 0.01$ and *** = $p < 0.001$. Y-axis labels are displayed in scientific notation where ae+b = $a \times 10^b$.

Table S1. Strain names and origin information table for *C. parapsilosis* strains.

Number	Name	Origin	Isolated from	Reference
CP1	CLIB214	Puerto Rico	Faeces	Type strain
CP2	CDC317	USA	Healthcare workers hand	Clark et al 2004 doi: 10.1128/JCM.42.10.4468-4472.2004

CP3	CDC173	USA	Blood or catheter cultures	Kuhn et al 2004 doi: 10.3201/eid1006.030873
CP4	711701	Aberdeen, UK	Unknown	Tavanti et al 2005 doi: 10.1128/JCM.43.1.284-292.2005
CP5	CDC167	USA	Blood or catheter cultures	Kuhn et al 2004 doi: 10.3201/eid1006.030873
CP6	J961250	Lisbon, Portugal	Nail	Tavanti et al 2005 doi: 10.1128/JCM.43.1.284-292.2005
CP7	CDC179	USA	Blood or catheter cultures	Kuhn et al 2004 doi: 10.3201/eid1006.030873
CP8	J930733	Beerse, Belgium	Cat hair	Tavanti et al 2005 doi: 10.1128/JCM.43.1.284-292.2005
CP9	103	London, UK	Anus	Tavanti et al 2005 doi: 10.1128/JCM.43.1.284-292.2005
CP10	J930631/1	Africa	Cat hair	Tavanti et al 2005 doi: 10.1128/JCM.43.1.284-292.2005

Table S2. Compound table with chromatographic and mass spectral validation

Retention time (min)	Compounds	Base Peak	NIST match score	Chemical formula	Molecular weight	Retention Index
**2.354**	Ethyl acetate	43	929	C4H8O2	88	888±8 (234)
**2.865**	Ethanol	45	946	C2H6O	46	932±8 (181)

**2.98**	Furan, 2,5-dimethyl-	96	950	C6H8O	96	939±9 (40)
**3.124**	Propanoic acid, ethyl ester	57	952	C5H10O2	102	953±7 (87)
**3.273**	Propanoic acid, 2-methyl-, ethyl ester	43	651	C6H12O2	116	961±6 (99)
**4.354**	Butanoic acid, ethyl ester	71	761	C6H12O2	116	1035±8 (251)
**4.464**	Methyl thiolacetate	43	895	C3H6OS	90	1052±5 (15)
**5.577**	1-Propanol, 2-methyl	43	776	C4H10O	74	1092±9 (269)
**5.774**	1-Butanol, 3-methyl-, acetate	70	945	C7H14O2	130	1122±7 (168)
**7.008**	Propanoic acid, pentyl ester	57	765	C8H16O2	144	1239±13 (12)
**7.649**	1-Butanol, 3-methyl-	55	909	C5H12O	88	1209±9 (375)
**8.493**	Styrene	104	788	C8H8	104	1261±10 (102)
**8.525**	3-Buten-1-ol, 3-methyl-	56	795	C5H10O	86	1248±8 (72)
**9.406**	Acetoin	45	895	C4H8O2	88	1284±12 (241)
**14.687**	2,3-Butandiol	45	956	C4H10O2	90	1565±18 (4)
**16.406**	1-Propanol, 3-(methylthio)-	106	891	C4H10OS	106	1719±9 (91)
**18.27**	Benzyl alcohol	108	836	C7H8O	108	1870±14 (323)
**18.649**	Phenylethyl alcohol	91	840	C8H10O	122	1906±15 (423
**19.885**	2-Pyrrolidinone	85	944	C4H7NO	85	2020±17 (7)
**19.976**	Nerolidol	69	918	C15H26O	222	2042±10 (172)

**21.674**	1H-Pyrrole-2,5-dione	97	821	C4H3NO2	97	
**22.455**	2,3-Dihydrofarnesol	69	797	C15H28O	224	2262±10
**22.941**	Farnesol	69	876	C15H26O	222	2323±19 (16)
**23.001**	trans-Farnesol	69	903	C15H26O	222	2356±10 (61)
**24.829**	Succinimide	99	866	C4H5NO2	99	2438±21 (2)
